# Analysis of sensorineural hearing loss and its relation to Optical Coherence Tomography Angiography findings in Behcet’s disease

**DOI:** 10.1007/s00405-025-09280-5

**Published:** 2025-03-18

**Authors:** Maha S. I. Abdelrahman, Mohamed G. A. Saleh, Maha Abdelgaber A. Aly, Shaimaa Salah

**Affiliations:** 1 Department of Rheumatology, Rehabilitation and Physical Medicine, Faculty of Medicine, Assiut University, Assiut, Egypt; 2 Department of Ophthalmology, Faculty of Medicine, Assiut University, Assiut, Egypt; 3 Audiovestibular Medicine unit, E.N.T Department, Faculty of Medicine, Assiut University, Assiut, Egypt

**Keywords:** Behcet’s disease, Optical coherence tomography angiography, Pure tone audiometry, Retinal vessels, Sensorineural hearing loss

## Abstract

**Objectives:**

We aimed to analyze auditory involvement in patients with Behcet’s disease and its association with Optical Coherence Tomography Angiography findings and disease manifestations.

**Methods:**

This study included 54 eyes and 68 ears of 34 adults with Behcet’s disease in comparison to 30 eyes and 60 ears of healthy controls. Clinical, laboratory and ophthalmological evaluation including Optical Coherence Tomography Angiography was done. Audiological assessment included otoscopic examination, immitancemetry and pure tone and speech audiometry.

**Results:**

Sensorineural hearing loss was observed in 32.35% of the studied cases. Behcet’s disease patients showed significantly higher average hearing level and significant reduction of retinal vessel density compared to controls. Patients with sensorineural hearing loss demonstrated significantly lower central retinal capillary vessel density and higher neuro-Behcet’s disease incidence in comparison to those without hearing loss (p-value = 0.039 and 0.024, respectively), which was confirmed by univariate regression analysis. After entering significant factors into the multivariate model, neuro-BD was identified as the most significant single predictor of sensorineural hearing loss in BD (p-value = 0.003).

**Conclusions:**

The association between sensorineural hearing loss and central retinal vessel density reduction and neuro- Behcet’s disease should be considered in Behcet’s disease.

## Background

Behcet’s disease (BD) is an inflammatory vasculitic disorder with a tendency to affect veins more than arteries [1]. It is a multisystem disease that may involve eyes, ears, joints, skin, central nervous system (CNS), gastrointestinal tract and lungs [2]. Generally, males have a more severe disease course than females [1]. It has a peculiar geographical distribution, being more frequent around the Mediterranean and Far East [3].

BD showed increased incidence of sensorineural hearing loss (SNHL) (12 to 80%) [4, 5]. Cochlear involvement is considered as a part of systemic vasculitic process on top of chronic inflammation [6]. Strong evidence for the role of humoral and T cell-mediated immunity in SNHL was proved [7].

Ocular involvement is the first manifestation of BD in about 10 to 20% of cases [8]. Ocular manifestations include uveitis (anterior, intermediate, posterior and panuveitis), optic neuropathy and retinal vasculitis [9]. Although, retinal vasculitis is the most frequent finding associated with uveitis involving the posterior segment, other vascular abnormalities such as ischemia and vascular occlusions can be detected [10]. Significant alterations in retinal and choroidal structure were observed in BD. Optical Coherence Tomography Angiography (OCTA) is a valuable tool in evaluating retinal vascular changes in BD [11].

So, both retinal vascular changes and hearing impairment in BD have a shared pathogenesis which is autoimmune vasculitis. Previous reports hypothesized a relation between SNHL and ocular ischemia in general population [12, 13]. This enhances our interest to investigate this relation in BD. While many studies had investigated auditory function [14, 15] and the ocular involvement in BD [16, 17], to date, no study had investigated the relation between auditory involvement and retinal vascular changes in BD. So, our study aimed to assess the auditory involvement in patients with BD, highlighting the importance of audiological screening and identifying potential association between hearing loss and retinal vascular abnormalities detected by OCTA and disease manifestations. Understanding this relation could improve patient care and education, follow up and quality of life as well as enhance exploring common mechanisms behind this potential association.

## Subjects and methods

### Ethical consideration

Study procedures followed the tenets of Helsinki declaration. Approval of the study by the local Institutional Review Board was obtained prior to the study, IRB no: 04-2024-300344. The study was conducted from January to July 2024. informed consent was obtained from all participants in the study.

### Subjects

This comparative cross-sectional study was carried out at the Rheumatology, Rehabilitation and Physical Medicine Department at Assiut University Hospital. The study conducted on 34 BD patients diagnosed according to the International Criteria for Behcet’s Disease [18] and 30 normal controls. The age of the participants ranged from 18 to 50 years.

Exclusion criteria were history of ototoxic drugs intake, any disease that may affect hearing function, ear trauma or surgery, significant media opacities in the eye and other ocular conditions that could cause irreversible posterior segment changes such as diabetic retinopathy, macular degeneration and optic nerve diseases such as glaucoma.

### Methods

Sociodemographic data, disease characteristics, and current medications use were collected for all included patients. Disease activity was evaluated using BD current activity form (BDCAF) which depends on the mucocutaneous, articular, gastrointestinal, neurological and chest symptoms present over the preceding 4 weeks [19]. Complete blood count, Erythrocyte sedimentation rate (ESR), liver and kidney functions were done.

Audiological evaluation was done at Audiovestibular Medicine unit, Assiut University hospital. All subjects underwent otoscopic examination to assess the external auditory canal and tympanic membrane. Basic audiological evaluation included immitancemetry, pure tone and speech audiometery was done.

Immitancemetry using Immitancemeter (Impedance Audiometer Interacoustic AT 235, Denmark) to evaluate middle ear function and measure the acoustic reflex by contralateral stimulation for frequencies 0.5, 1, 2 and 4 kHz. Pure tone audiometry using dual channel clinical audiometer (Orbiter 922, GN Otometrics, Cobenhagen, Denmark) was performed to evaluate: air conduction pure tone thresholds, for each ear separately, at octave frequencies 0.25–8 kHz and bone conduction pure tone thresholds, for each ear separately, at octave frequencies 0.5–4 kHz. Speech audiometry using dual channel clinical audiometer (Orbiter 922) was performed to assess speech reception threshold test for each ear separately using spondee words list [20] and speech discrimination score for each ear separately using Arabic phonetically balanced words [21].

Degree of hearing level was identified by calculating the average of pure tone audiometery at 500,1000,2000 and 40,000 Hz as follows: normal (equal or less than 25 dB) mild hearing loss (26 to 40 dB), moderate hearing loss (41 to 55 dB), moderately severe hearing loss (56 to 70 dB), severe hearing loss (71 to 90 dB) and profound hearing loss (91 + dB) [22].

Ophthalmological examination was done at department of Ophthalmology, Assiut University hospital. A uveitis specialist performed clinical examination (including assessment of Snellen’s best-corrected visual acuity, slit-lamp examination, applanation tonometry, and dilated fundus examination with noncontact or contact lenses) and imaging of subjects’ eyes (this included fundus fluorescein angiography (FFA) and OCTA). Active uveitis was identified as presence of vitritis, retinal vascular sheathing or leakage on FFA, retinal infiltrates, optic disc edema, or anterior uveitis based on SUN (standardization of uveitis nomenclature) criteria [23].

OCTA was performed via a commercially available swept-source OCT (SS-OCT) device (DRI OCT Triton plus; Topcon) which operates at 100,000 A-scans per second using a light source of 1,050 nm. All scans were acquired over a 4.5 × 4.5 mm and a 6 × 6 mm field of view. En-face images of both the superficial capillary plexus (SCP) and the deep capillary plexus (DCP) were obtained. We excluded poor quality OCT angiograms with motion artifacts. The 4.5 × 4.5 mm OCT angiograms of the SCP and DCP were qualitatively analysed for the following: perifoveal anastomotic capillary arcade disruption in the SCP, capillary morphological changes and areas of capillary nonperfusion/hypoperfusion. Quantitative assessment, done via built-in software in the OCT device (IMAGEnet 6 version 1.22.1.14101), included foveal avascular zone (FAZ) area and capillary vessel density (VD) measurement in SCP, calculated as the percentage of pixels occupying the screen representing flow. FAZ area was manually defined and measured in mm^2^. The measurements were averaged to get a final estimate, then compared with those obtained in healthy matched subjects.

### Statistical analysis

Sample size was calculated via open Epi V.3.01 software. Data analysis was performed using SPSS version 26. Numerical data was expressed as mean ± SD for normally distributed data or median (range) for non-normally distributed data. Chi square and Fisher´s exact tests were used to compare proportions. Student T test and Mann -Whitney U test were used to compare mean/ mean rank among groups. For correlations, we applied Pearson’s or Spearman’s correlations. Univariate and multivariate logistic regression analyses were utilized to identify factors associated with SNHL among BD patients. The explanatory variables entered in the adjusted multivariate regression models were the significant variables resulting from the univariate regression analysis. P value was considered significant at less than 0.05.

## Results

The current study was conducted on 54 eyes and 68 ears of 34 BD patients in comparison to 30 eyes and 60 ears of 30 control subjects. The mean age of the included cases was 34.12 ± 6.92 versus 31.43 ± 5.11 years for healthy subjects (p-value = 0.09). Most of the included participants were males; 27 cases (79.41%) and 25 controls (83.33%) (p-value = 0.688). The median disease duration of the study cases was 5.5 years (ranged from 1 to 17 years). The most common symptom at the time of the study was oral ulcers (47.06%). Other symptoms included genital ulcers (20.59%) and articular manifestations (11.76%). Our results indicated that 8 patients (23.53%) had auditory symptoms in the form of decreased hearing and tinnitus. Detailed clinical and laboratory data are displayed in Table 1.

**Table 1:** Clinical and laboratory characteristics of BD patients

Parameter Mean ± SD/median(range) or *n* (%)	BD patients (*n* = 34)
Disease duration (years) median (range)	5.5 (1–17)
Oral ulcers	16 (47.06%)
Genital ulcers	7 (20.59%)
Articular symptoms	4 (11.76%)
History of thrombosis	13 (38.24%)
BDCAF median (range)	2 (0–8)
Auditory symptoms	8 (23.53%)
History of neuro- BD	7 (20.59%)
Ocular involvement (anterior and posterior segments and retinal vasculitis)	
• Active	16 (47.06%)
• Inactive	18 (52.94%)
**Laboratory investigations**	
• White blood cell count (x10³/mm³)	7.07 ± 2.26
• Hemoglobin (gm/dl)	13.78 ± 1.67
• Platelets (x10³/µl)	292.15 ± 80.16
• Aspartate transaminase (U/L)	20.09 ± 5.41
• Alanine transaminase (U/L)	18.9 ± 6.5
• Blood urea nitrogen (mmol/L) median (range)	3.9 (2.6–6.5)
• Serum creatinine (µmol/l)	67.28 ± 13.19
• First hour ESR (mm/hr) median (range)	17.5 (2-100)

Data expressed as mean ± SD / median (range) or number (frequency %). BDCAF: Behcet’s disease current activity form, ESR: erythrocyte sedimentation rate

The most frequently administrated medication by the study patients was prednisolone (taken by 58.82% of the patients) followed by methotrexate (50%), colchicine (44.12%), azathioprine (38.24%), adalimumab (20.59%), cyclophosphamide (11.76%), warfarin (8.82%), mycophenolate mofetil (2.94%), and cyclosporine (2.94%).

**Fig. 1:**
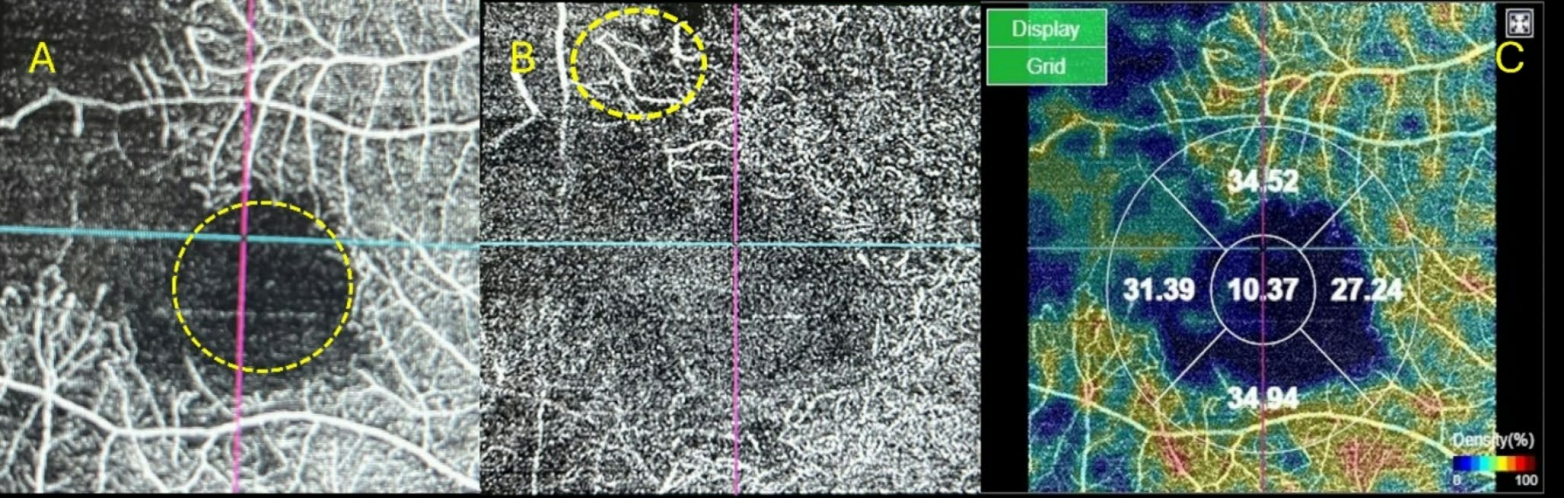
OCTA angiography finding. **A**: 4.5 × 4.5 mm OCTA scan of superficial capillary plexus of right eye of a male patient with BD showing extensive areas of capillary dropout and enlargement of the foveal avascular zone (yellow dashed circle). **B**: 4.5 × 4.5 mm OCTA scan of deep capillary plexus showing telangiectatic capillaries (corkscrew appearance) (yellow dashed circle). **C**: OCTA vessel density map. OCT-A images depict vessels through different segmented layers of the retina as the superficial retinal plexus (large radial linear vessels converging towards the fovea) (seen in Fig. **A**) and the deep retinal plexus (network of fine interwoven plexus concentrically arranged around the fovea) (seen in Fig. **B**). This arrangement produces a capillary-free region seen as black area referred to as the FAZ which is very sensitive to ischemic changes showing enlargement and irregular outline. Ischemic areas within the retina show reduced number of capillaries against a greyish background (capillary dropout). Vascularity of the retina can be quantitatively expressed using Vessel Density (a unitless measure that reflects the proportion of the image occupied by vessels which can be measured as proportion of the white pixels divided by the total pixels in the image) which is shown on a map for different quadrants of the retina as seen in Fig. **C**

BD patients showed significantly higher average hearing level in comparison to healthy participants (p-value < 0.001). In addition, OCTA examination of studied cases showed significant retinal ischemia compared to healthy controls as shown in Fig. 1. Retinal capillary VD and FAZ are demonstrated in Table 2.

**Table 2:** OCTA and the average hearing level of studied cases in comparison to healthy controls

Parameter mean ± SD/median(range)	BD cases (*n* = 54 eyes, 34 right ears and 34 left ears)	Control eyes (*n* = 30 eyes, 30 right ears and 30 left ears)	*P* value
Nasal VD	43.89 ± 4.06	47.27 ± 1.75	**< 0.001**
Temporal VD	44.61 ± 4.14	48.92 ± 2.18	**< 0.001**
Superior VD	46.45 ± 5.58	50.09 ± 2.22	**< 0.001**
Inferior VD	45.7 ± 5.81	49 ± 2.31	**0.002**
Central VD	20.13 ± 7.17	23.63 ± 3.47	**0.011**
FAZ (µm^2)^	636.5 (124.45-4622.7)	256.1 (136.67-415.11)	**< 0.001**
Average hearing level of right ear	19. 38 (12.5-43.75)	15 (10–20)	**< 0.001**
Average hearing level of left ear	17.5 (12.5–35)	15 (11.25–22.5)	**< 0.001**

Data expressed as mean ± SD / median (range). P-value is significant at < 0.05

BD: Behcet’s disease, VD: vessel density, FAZ: foveal avascular zone

We observed that 32.35% of the studied cases (11 cases) had SNHL; 7 cases had unilateral SNHL while 4 cases had bilateral SNHL as illustrated in Table 3. However, 7 out of those 11 cases did not show any auditory complaints. Eight patients had high frequencies hearing loss. The remaining 23 cases had within normal average hearing level however, we observed significant difference between those cases and control group regarding hearing level (17.5 (11.25-25) versus (15 (10–20)) *p* < 0.001 respectively for the right ear and 16.25 (11.25–22.5) versus 15 (11.25–22.5) *p* < 0.001 respectively for the left ear).

**Table 3:** Laterality and the degree of hearing loss in the study group

Parameter	*N* = 34 (%)
**SNHL**	11 (32.35%)
• Unilateral	7 (20.59%)
• Bilateral	4 (11.76%)
**Severity of SNHL**	
• Mild	6 (17.65%)
• Moderate	4 (14.71%)
• Moderately severe	1 (2.94%)
• Severe	0 (0%)
• Profound	0 (0%)

Data expressed as number (frequency %). SNHL: sensorineural hearing loss

BD cases were divided into 2 groups according to presence or absence of SNHL. No significant association could be detected between SNHL and clinical and therapeutic features of studied cases (age, sex, disease duration, active eye involvement, neuro-BD, history of thrombosis and therapeutic history) except for neuro-BD (p-value = 0.024). In addition, we found that patients with SNHL demonstrated significantly lower central retinal capillary VD in comparison to those without hearing loss (p-value = 0.039). These findings were consistent with the univariate regression analysis. Neuro-BD and central retinal VD were entered into the multivariate model. The history of neuro-BD was identified as the most significant single predictor of SNHL in BD as shown in Table 4.

**Table 4:** Factors associated with SNHL in BD

Predictors	Univariate		Multivariate	
	OR (95% CI)	*P*-value	AOR (95% CI)	*P*-value
Age	0.999 (0.899–1.11)	0.987		
Male gender	0.283 (0.03–2.706)	0.273		
Disease duration	1.006 (0.865–1.17)	0.936		
BDCAF	0.937 (0.622–1.411)	0.756		
Neuro-BD	**0.114 (0.018–0.745)**	**0.023**	**0.098 (0.021–0.455)**	**0.003**
Biological treatment	1.25 (0.202–7.75)	0.811		
Nasal VD	0.921 (0.798–1.061)	0.254		
Temporal VD	0.918 (0.787–1.071)	0.275		
Superior VD	1.015 (0.961–1.125)	0.773		
Inferior VD	0.973 (0.882–1.074)	0.59		
Central VD	**0.903 (0.818–0.997)**	**0.043**	0.903 (0.806–1.012)	0.078
FAZ	1.001 (1-1.001)	0.093		

Logistic regression analysis, Background LR model. P-value is significant at < 0.05

OR (odds ratio), AOR (adjusted OR), 95% CI (95% confidence interval)

BD: Behcet’s disease, SNHL: sensorineural hearing loss, BDCAF: Behcet’s disease current activity form VD: vessel density, FAZ: foveal avascular zone

No significant correlation could be detected between right and left average hearing level on one side and age, disease duration, BDCAF and ESR on the other side as illustrated in Table 5.

**Table 5:** Correlation study of right and left average hearing level with different variables

Variable	Average hearing level of the right ear		Average hearing level of the left ear	
	*R*	*P*	*R*	*P*
Age	-0.011	0.951	0.07	0.695
Disease duration	-0.04	0.823	0.162	0.361
BDCAF	-0.220	0.211	0.06	0.734
ESR	0.175	0.324	0.168	0.342

P value is significant at < 0.05. R: correlation coefficient, E.S.R: erythrocyte sedimentation rate, BDCAF: Behcet’s disease current activity form

## Discussion

To our knowledge, this is the first study to investigate a possible relation between SNHL and retinal vascular changes in BD. Our study revealed a significantly lower central retinal capillary VD and higher incidence of neuro-BD in BD patients with SNHL compared to those without hearing loss.

Manifestations of BD can affect any body organ, as it involves different blood vessels regardless of their size [24]. Audio vestibular involvement in BD was first reported by Alajouanine et al. [25]. Hearing loss in BD was proved to be the third or fourth most frequent clinical finding [15, 26, 27]. In our study, it emerged as the third most common finding following oral ulcers and ocular involvement. SNHL was detected in 23% of studied BD cases by Sonbay et al. [5], in 9.3% by Bayraktar et al. [28]. In our study, SNHL was observed in 11 cases (32.35%). However, only 4 patients out of 11 expressed an auditory complaint as most of them had mild to moderate SNHL. This suggests that all BD patients should be screened for hearing impairment. We observed that 8 patients out of 11 (72.73%) had a high frequencies hearing loss, and this is consistent with the previous studies which reported that hearing loss exists in autoimmune disorders, commonly at high frequencies _[5, 26, 28]_. In addition, Erdinc et al. observed that 68% of BD patients with hearing loss had high frequencies hearing loss [27].

OCTA is a non-invasive imaging tool for assessing the microvascular structures in the eye without dye [29]. Our findings showed that VD in different retinal areas was significantly lower in BD patients compared to healthy controls. This comes in agreement with Koca et al. who found significant reduction in retinal VD in ocular BD patients in comparison to healthy controls (p-value < 0.001) [30]. The reduction in VD can be attributed to diffuse occlusive vasculitis which is common in BD [31].

As an indication of a high degree of vascular impairment and capillary hypoperfusion around the fovea, BD patients demonstrated a greater FAZ diameter compared to healthy controls (p- value < 0.001). These findings were consistent with the previous reports [32–34]. Koca et al. could not detect significant difference between the BD group and control group regarding FAZ size (*p* = 0.266) however, they observed significant differences in perifoveal capillary hypoperfusion, perifoveal capillary network disorganization and FAZ irregularity between ocular and non ocular BD patients (p-value < 0.001) [30]. The variability between studies may be explained by differences in age, severity of the disease, number of previous ocular attacks and availability of efficient treatment modalities.

Neuro-BD is one of the most serious complications of BD. The incidence of neuro-BD shows great variability worldwide. It is observed in approximately 10–30% of patients with BD. This great variability is attributed to certain factors such as type of study, ethnicity and therapeutic protocols [35]. A retrospective study of 96 BD patients reported 24% incidence rate of neuro- BD [36] which is similar to our results as 20.59% of our study cases were diagnosed with neuro-BD. In a cohort of 113 patients diagnosed with BD, 13.4% of the included patients had neuro-BD [37].

Based on our study findings, no association could be detected between SNHL and clinical or therapeutic features of studied cases except for neuro-BD and central retinal capillary VD. A previous study included 63 BD patients did not find an association between SNHL and other system involvement including neurological system. This disagreement with our results could be explained by low percentage of BD patients with neurological involvement in the previous study (6.35%) [27]. Sota et al. observed that CNS manifestations were present in 64% of BD patients with SNHL vs. 50% in those without SNHL (*p* = 0.525) [15].

The association between SNHL and neuro-BD could be attributed to a shared pathological insult which is vasculitis of the origin of vestibulocochlear nerve in the brainstem. Previous reports concluded that BD may be presented with small vessel vasculitis, frequently affecting the brain stem which may result in subacute cranial neuropathy. In addition, they indicated that central vestibular tract involvement may denote concealed brain stem involvement [38–40]. We observed that patients with SNHL demonstrated significantly lower central retinal capillary VD in comparison to those without hearing loss (p-value = 0.039) which may be explained by that both are attributed to small vessel vasculitis. Kim et al. reported an increased incidence of retinal occlusive vasculopathy in SNHL patients in general population compared to matched controls supposing that both conditions share common risk factors and etiopathogenesis [13]. Another study investigated the incidence of hearing loss in patients with ocular ischemic disorders, but no significant association was detected, however this study depended on investigating hearing loss via an interview ( United States National Health Interview Survey data of hearing loss by National Center for Health Statistics) and not on audiometry tests [12].

Sota et al. reported a significant association between SNHL and combined cutaneous and articular involvement (*p* = 0.013). However, detached analysis of articular and skin manifestations did not yield significant differences (*p* = 0.085 and *p* = 0.067) [15].

In agreement with Pollak et al. [41], our results indicated that age and disease duration did not show significant impact on average hearing level. Two studies reported that there was no correlation between hearing loss and duration of the disease however, they observed that BD patients with SNHL were significantly older than those without hearing loss. The mean age of the included BD patients by Erdinc et al. and Evereklioglu et al. was 35 ± 8.73; (range, 15–63) and 34.96 ± 8.50 years, respectively [27, 42]. Both studies did not exclude patients older than 50 years. In our study, patients aged more than 50 years were excluded to avoid the confounding effect of age on auditory function and this may explain the difference between our results and results of the aforementioned studies.

Our study aimed to reduce the likelihood of confounding by establishing the previously mentioned exclusion criteria and comparing patients with and without SNHL regarding different parameters that could constitute a confounding factor for hearing loss such as medications.

## Conclusions and recommendations

Hearing loss is a common underestimated issue in BD and should be kept in mind, so it may be beneficial to include hearing tests in routine follow-up visits especially high frequencies to detect early hearing loss. OCTA is a safe noninvasive method for assessing retinal vascular involvement in BD. In our study, we observed a relation between hearing loss and both neuro-BD and central retinal VD reduction so this association should be considered in BD patients. Given the complexity of the relationship between age, auditory function and retinal vascularity, future research with a larger sample size and a longitudinal component is crucial. This will help understand causality and temporal relationship between SNHL and retinal ischemia in BD patients. Moreover, investigating cochlear and vestibulocochlear vasculitis in BD patients with active retinal vasculitis will be valuable in assessing the potential association between these conditions and explaining the pathophysiologic mechanism of this association if present.

### Limitations

The current study has some limitations like the limited sample size due to presence of multiple exclusion criteria and noncompliance of some patients with all study procedures. In addition, the cases were recruited from a single center.

## Data Availability

Data generated or analysed during this study are included in this published article and available from the corresponding author on reasonable request.

## Abbreviations

BD: Behcet’s disease
BDCAF: Behcet’s disease current activity form
CNS: Central nervous system
dB: Decibel
DCP: Deep capillary plexus
ESR: Erythrocyte sedimentation rate
FAZ: Foveal avascular zone
FFA: Fundus fluorescein angiography
OCTA: Optical Coherence Tomography Angiography
SCP: Superficial capillary plexus
SNHL: Sensorineural hearing loss
SUN: Standardization of uveitis nomenclature
VD: Vessel density
